# How perceptual learning rewires brain connectivity: lessons from the visual system in a top-down perspective

**DOI:** 10.3389/fncir.2025.1636023

**Published:** 2025-08-25

**Authors:** Alan Consorti, Alessandro Sale

**Affiliations:** ^1^F.M. Kirby Neurobiology Center, Department of Neurology, Boston Children’s Hospital, Harvard Medical School, Boston, MA, United States; ^2^Neuroscience Institute, National Research Council (CNR), Pisa, Italy

**Keywords:** perceptual learning, neural plasticity, visual processing, circuit rewiring, top-down integration

## Abstract

Neural circuits sculpt their structure and modify the strength of their connections to effectively adapt to the external stimuli throughout life. In response to practice and experience, the brain learns to distinguish previously undetectable stimulus features recurring in the external environment. The unconscious acquisition of improved perceptual abilities falls into a form of implicit learning known as perceptual learning. Despite more than a century of multidisciplinary studies, a thorough understanding of the neural mechanisms underlying perceptual learning is still missing. Increasing evidence suggests that the learning process induces global plastic remodeling across several cortical areas, tuning neural responses to changing environmental claims by reweighting the interaction between bottom-up and top-down information. Here, we will survey classic and novel findings in the field of perceptual learning research, with a particular focus on visual perceptual learning.

## 1 Introduction: neural plasticity foundations of perceptual learning

The lifelong enhancement of perceptual abilities in response to experience represents a specific type of implicit learning known as perceptual learning (PL) (Gibson, 1969). PL is an adaptive process that involves relatively long-lasting changes in the perceptual systems, improving the organism’s ability to respond to specific external features that recur in its environment (Goldstone, 1998). A large body of studies shows that PL occurs in all sensory modalities and in response to a plethora of different perceptual tasks. These tasks can range from simple discriminations to complex categorizations. Simple tasks include discrimination of visual orientation (e.g., Schoups et al., 1995; Shiu and Pashler, 1992; Vogels and Orban, 1985), auditory pitch (e.g., Recanzone et al., 1993; Wright and Fitzgerald, 2001), and tactile frequency (e.g., Jenkins et al., 1990; Recanzone et al., 1992). Complex tasks include discrimination of objects, complex forms, and recognition of faces (e.g., Fine and Jacobs, 2000; Furmanski and Engel, 2000; Hussain et al., 2009; Mclaren, 1997).

In this review, we provide an updated and integrative perspective on how perceptual learning reshapes brain connectivity, with a particular focus on the visual system. While previous literature has extensively documented plastic changes in lower-order brain regions, our review highlights emerging evidence that underscores the critical role of top-down interactions in driving functional reorganization across cortical networks. Departing from traditional approaches that primarily emphasize early sensory areas and bottom-up mechanisms, we discuss how perceptual learning reflects a dynamic reweighting between ascending and descending signals within the visual hierarchy.

A key novelty of this review lies in its system-level approach: rather than focusing solely on localized neuronal tuning, we examine how experience-dependent changes affect connectivity between hierarchically distinct visual areas. This broader perspective integrates findings across multiple levels of analysis - ranging from microcircuit modifications to large-scale network dynamics - and considers their implications for cognition and neural plasticity.

The temporal scope of the reviewed literature spans from 1970 to 2025, including peer-reviewed original research articles, systematic reviews, and meta-analyses identified through searches in academic databases such as PubMed, Scopus, and Web of Science.

### 1.1 PL and circuit-wide rewiring

Although the precise mechanisms underlying PL remain unclear, it is currently held that perceptual improvements rely on network rearrangements that lead to circuit-wide rewiring in the adult brain.

Brain wiring emerges from the interaction between intrinsic genetic pathways and the response to environmental stimuli, a complex interplay taking place during specific developmental windows known as critical periods (CPs), when neural plasticity is considerably enhanced. As the brain matures, CPs close, leading to a pronounced decrease in plasticity, likely driven by evolutionary pressures to consolidate adult neuro-connectivity, in order to maintain brain functions acquired during development (Berardi et al., 2000; Hensch, 2005).

Nonetheless, neural connections retain an intrinsic reservoir of plasticity throughout the lifespan, preserving a certain degree of adaptability essential for proper neural functions in an ever-changing environment (Baroncelli et al., 2010; Consorti et al., 2019). The adult neuroarchitecture can therefore be rewired even far beyond the closure of CPs, although in a much more specific and local manner (Bavelier et al., 2010; Fagiolini and Hensch, 2000; Morishita et al., 2010; Sansevero et al., 2019). Even highly organized and rigid sensory maps can be modified by experience in adulthood (Xerri, 2012; Irvine, 2018; Quentin et al., 2019). For instance, the somatosensory body representation in the adult brain can be re-mapped after peripheral lesions. After a few weeks, the cortical region corresponding to the lesion can become responsive to stimulation of neighboring regions of the skin (Merzenich et al., 1984). Appreciable changes in the somatosensory map can also occur in response to motor experience: the cortical representation of a digit used to repeatedly perform a given task can expand at the expense of other digits (Jenkins et al., 1990). In the visual system, neurons within a cortical scotoma regain their responsiveness by shifting their tuning outside the injured region within 2 months after the injury (Gilbert, 1992). Historically, the unexpected plastic remodeling observed in adult sensory cortices has led to the hypothesis that analogous changes might underlie PL under physiological conditions (Gilbert et al., 2001).

Pioneering studies on the cellular mechanisms underlying PL initially associated this form of learning with topographic reorganizations within the trained brain regions. The cortical area within the sensory cortex that represents the trained stimulus parameter can expand its territory by recruiting adjacent untrained regions. Typical examples are cortical changes observed in the monkey somatosensory and auditory cortices in response to perceptual practice (Recanzone et al., 1992, 1993). For instance, training in a vibration frequency discrimination task leads to a cortical expansion of the neural representation of the trained skin area within the primary somatosensory cortex (Recanzone et al., 1992; Pleger et al., 2003; Dempsey-Jones et al., 2015; Pacchiarini et al., 2017; Chéreau et al., 2020). Similarly, training in acoustic discrimination enlarges the cortical area representing the trained frequencies within the primary auditory cortex (Recanzone et al., 1993; Polley et al., 2006; Irvine, 2018; Bajo et al., 2019; Maor et al., 2019; Grootjans et al., 2023).

PL-induced cortical changes may also be encoded by changes in the temporal firing patterns of neurons (Gilbert et al., 2001), modulating the synchrony of the response in a neuronal population. Learning can, for instance, synchronize cell firing to the trained stimulus (Mountcastle et al., 1990), with the neuronal subpopulations acquiring a high temporal coherence with the stimulus when trained in a frequency discrimination task (Recanzone et al., 1993). Temporal training discrimination can remarkably alter cortical response dynamic in the primary auditory cortex (Bao et al., 2004).

Cortical recruitment and temporal changes in the firing pattern have been mainly observed in somatosensory and auditory cortices. Visual perceptual learning (vPL), on the other hand, seems not to rely on major topographic reorganizations, but preferentially on functional plastic changes in the response properties of visual neurons. Improvements in the behavioral performance of monkeys trained in an orientation discrimination task were shown to be correlated with changes in the orientation tuning of primary visual cortex (V1) neurons (Schoups et al., 2001), rather than expanding the topographic representation of trained visual neurons. An increase in orientation selectivity was only present within the retinotopic region subjected to the orientation discrimination practice; no significant changes were observed in untrained neurons, suggesting that the neuroplastic changes induced by vPL are selective for the features of the trained stimuli (Schoups et al., 2001). Using 2-photon calcium imaging, tuning curve plasticity was similarly detected in the specific subset of V1 neurons with a preferred orientation close to the training stimulus (Schumacher et al., 2022). It seems, then, that vPL relies on completely distinct circuit changes compared to what was found in the somatosensory and auditory areas.

**FIGURE 1:**
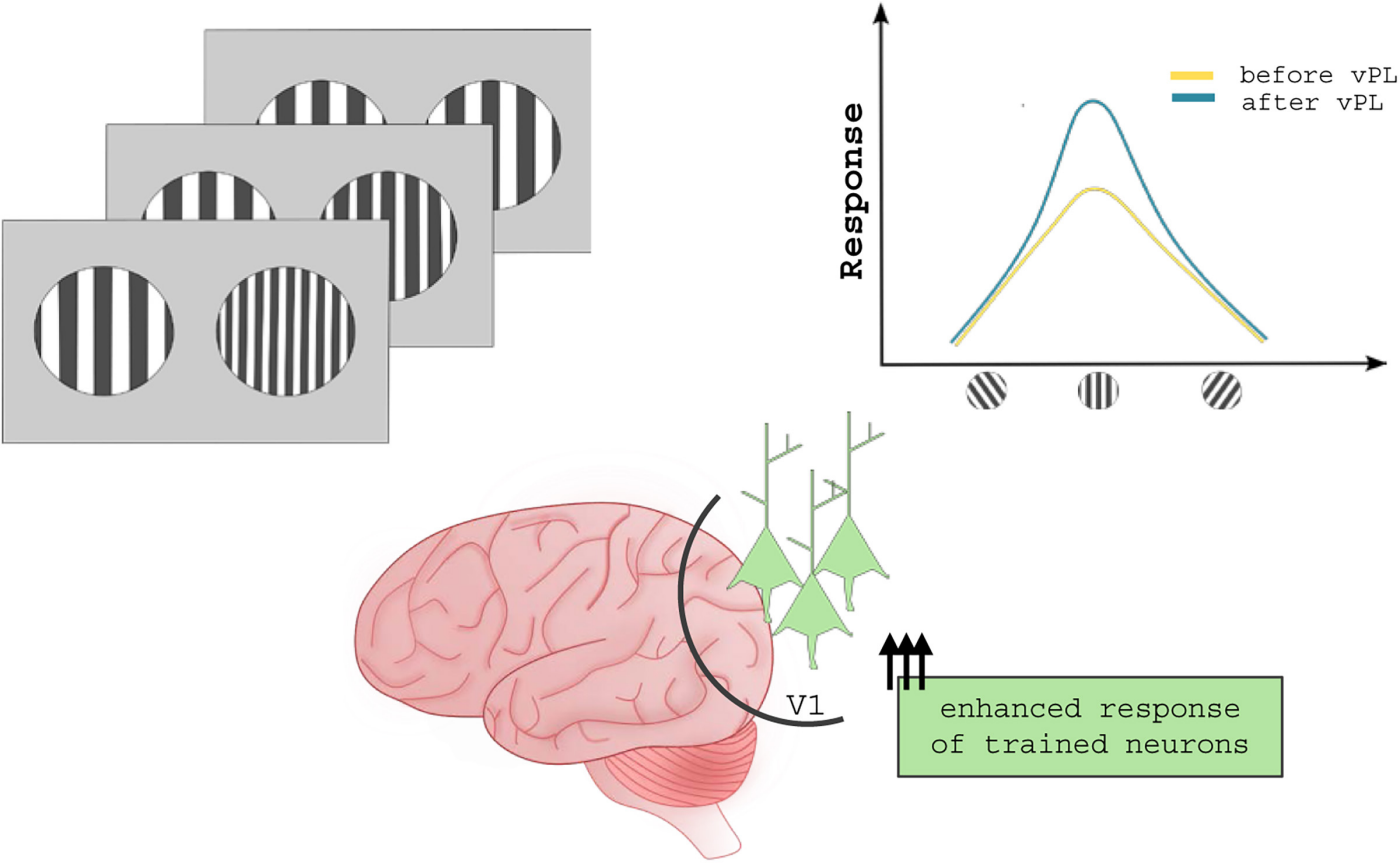
Neuronal mechanisms underlying vPL. Several studies suggest that vPL enhances response tuning of those neurons coding for task-relevant features, ultimately improving the signal/noise ratio. In contrast to what was described for sound and tactile practice, vPL changes the activity but not the topography of visual cortical areas.

While the great majority of studies focus on PL-induced modifications within V1 circuitry, changes in orientation tuning curves were also observed in higher-order areas. For example, training in an orientation match-to-sample task can tune the activity of neurons lying in the visual area 4 (V4) of rhesus monkeys (Yang and Maunsell, 2004). Notably, vPL can induce larger changes in the response properties of V4 neurons than those found in V1 (Raiguel et al., 2006). According to several theories, vPL can consolidate the cortical representation of trained stimuli by increasing the weight of task-relevant features at the expense of task-irrelevant information (Figure 1). Visual neurons, in this view, act as adaptors whose response can be modulated by contingent environmental needs: a specific neuron in the visual circuit has a certain degree of freedom in selecting the stimulus feature to represent, rather than having a fixed selectivity for a given stimulus component (Astorga et al., 2022).

## 2 Top-down modulation in vPL

The visual system stands as one of the prime models to study PL, mainly for the extensive body of research and knowledge accumulated on this sensory system over the last decades. vPL has been documented in response to discrimination of orientation (Karni and Sagi, 1993; Matthews et al., 1999; Matthews and Welch, 1997; Schoups et al., 1995; Shiu and Pashler, 1992; Vogels and Orban, 1985) motion direction (Ball and Sekuler, 1982, 1987), texture (Karni and Sagi, 1991; Ahissar and Hochstein, 1996) differences in the waveform between two sinusoidal stimuli (Berardi and Fiorentini, 1987; Fiorentini and Berardi, 1980; Fiorentini and Berardi, 1981), detection of visual gratings (De Valois, 1977; Mayer, 1983), changes in spatial frequency within simple or complex plaid patterns (Fine and Jacobs, 2000), ability to detect small depth differences between two targets (Fendick and Westheimer, 1983; Westheimer and Truong, 1988) or to perceive depth in random-dot stereograms (Ramachandran and Braddick, 1973), and object (Furmanski and Engel, 2000) and face (Gold et al., 1999) recognition.

Visual perceptual learning is considered a long-lasting phenomenon. Consistently, perceptual improvements in visual perception can persist from months to years (Ball and Sekuler, 1987; Fiorentini and Berardi, 1981; Karni and Sagi, 1993). Once learned, the improved discriminability can be retrieved after years without the need of further training, even after a prolonged period of inactivity (Karni and Sagi, 1993). However, the time course required to acquire such expertise seems to be task specific. For some tasks, vPL occurs within one or two hours of training, showing a fast saturation after few hundred of trials (Fahle et al., 1995; Fiorentini and Berardi, 1980; Fiorentini and Berardi, 1981; Liu and Vaina, 1998; Shiu and Pashler, 1992). For other tasks, vPL is characterized by a fast phase of saturation followed by a slow phase during which the performance improves from one daily session to the next, until a stable plateau is reached (Karni and Sagi, 1991). For some other tasks, instead, vPL occurs after thousands of trials or even after years, displaying the characteristics of a long-term learning process (Karni and Sagi, 1993). Interestingly, studies have shown that the number of sessions needed to observe perceptual improvement can be reduced by combining vPL with transcranial electrical stimulation (Fertonani et al., 2011; Maniglia, 2022). In particular, alpha oscillations can enhance the learning rate and performance improvement of vPL during orientation discrimination (He et al., 2022, 2023).

A controversial and disputed issue in vPL research is the specificity of the acquired perceptual improvements. Traditional studies suggest that vPL is highly specific for the features and location of the stimuli employed during perceptual practice. When simple stimuli are involved, the perceptual improvement obtained by practicing the discrimination task is usually lost when the trained stimulus parameters are changed. Specificity of vPL has been widely documented for orientation (Fahle and Edelman, 1993; Fiorentini and Berardi, 1980; Fiorentini and Berardi, 1981; Karni and Sagi, 1991; McKee and Westheimer, 1978; Poggio et al., 1992; Ramachandran and Braddick, 1973; Schoups et al., 1995), spatial frequency (Fiorentini and Berardi, 1980; Fiorentini and Berardi, 1981), motion direction (Ball and Sekuler, 1982, 1987), chromatic contrast, and for the location of the stimulus in the visual field (Ball and Sekuler, 1987; Fiorentini and Berardi, 1981; Karni and Sagi, 1991; Schoups et al., 1995; Shiu and Pashler, 1992).

Other studies, however, suggest that vPL can display various degrees of transfer properties under several conditions, especially when more complex stimuli are involved (McGovern et al., 2012; Awada et al., 2021). Thus, specificity versus learning transfer seems to depend on multiple factors, such as the level of the trained task (Fine and Jacobs, 2000), task difficulty (Ahissar and Hochstein, 1997), precision of the transfer task (Jeter et al., 2009), the extent of training (Jeter et al., 2010), state of adaptation induced by training (Censor et al., 2006), the exact training and transfer procedures (Xiao et al., 2008; Hung and Seitz, 2014), attention (Hung and Carrasco, 2021), and even stimulus feature variability (Manenti et al., 2023).

### 2.1 Cortical localization of vPL

One of the most controversial issues in vPL research is defining which cortical stage of visual processing accounts for the acquisition of perceptual improvements. Historically, the residual levels of neural plasticity observed in fully developed early visual areas have led to the hypothesis that the cortical changes underlying vPL are localized early along the visual pathway. In this scenario, vPL relies mainly on feedforward (bottom-up) inputs streaming from the retina. Consistently, neuronal changes associated with vPL have been documented in several visual areas (Pourtois et al., 2008; Bao et al., 2010; Yu et al., 2016). The specificity of vPL for simple stimulus features, as the trained orientation and position (Karni and Sagi, 1991), has been moreover taken as indirect proof supporting the centrality of early visual areas in visual perceptual practice.

However, despite the vPL specificity for stimulus parameters, it is unlikely that feedforward connections alone can fully account for the complexity of vPL. Accumulating studies have now started to document vPL-induced changes in higher stages of the visual pathway, suggesting that higher-order visual areas are crucially implicated in vPL (Zohary et al., 1994; Yang and Maunsell, 2004; Gold et al., 2008; Adab et al., 2014). Then, the observations of learning transfer of perceptual improvements (McGovern et al., 2012; Awada et al., 2021) have suggested that vPL requires at least the activation of higher visual stages, where neurons are less specific for simple stimulus features (Smith et al., 2001). Nonetheless, testing the direct involvement of top-down processing in vPL remained elusive for decades, largely due to the lack of advanced techniques capable of investigating whether plastic changes in higher-order visual areas arise through purely feedforward mechanisms or are correlated with the activation of feedback projections.

#### 2.1.1 Changes in lower-order visual areas

Classic physiological and psychological observations designate V1 as a central hub in vPL. V1 neurons have smaller receptive fields than those located in higher visual cortices (Goldman-Rakic and Rakic, 1991) and are selective for simple stimulus features (Hubel and Wiesel, 1959). The neurophysiological properties of V1 neurons, therefore, can account for the specificity of the trained location, feature, and eye associated with perceptual practice. Consistently with this view, several experiments reported that vPL correlates with V1 structural changes in both human and non-human primates.

In humans, a pivotal study showed that improvements in a motion-detection task correlate with changes in the visual representation of V1 neurons (Fahle and Skrandies, 1994). Using multichannel evoked-potentials, the authors recorded significant differences in potential distributions with a latency of 100 ms over the occipital lobe, a result that suggests the involvement of human V1 in vPL (Fahle and Skrandies, 1994). Supportive evidence comes from electrophysiological and functional imaging studies. Changes in an early visual area response have been found after practice of different tasks measuring C1, the earliest component of the visual evoked potentials (Pourtois et al., 2008; Bao et al., 2010). These electrophysiological changes have been also associated with retinotopic changes occurring in V1 (Furmanski et al., 2004; Hua et al., 2010; Jehee et al., 2012). In addition, an increase in blood-oxygenation-level-dependent (BOLD) signal in the trained V1 region has been associated with vPL (Yotsumoto et al., 2008). Lastly, vPL has been shown to even affect pre-cortical circuits: a contrast detection vPL task has indeed shown to correlate with neuronal response changes in the M-layers of the lateral geniculate nucleus (Yu et al., 2016).

In non-human primates, electrophysiological studies have shown that vPL for simple discrimination tasks or contour-detection tasks alter neuronal response properties in V1 (Crist et al., 2001; Schoups et al., 2001; Li et al., 2008). Using chronically implanted multielectrode arrays, the dynamic changes in the response properties of V1 neurons were captured over the course of a contour detection task (Yan et al., 2014). The results showed a progressive strengthening in the facilitation of neurons encoding trained contour elements, with suppression of neurons responding to background components (Yan et al., 2014). Practicing a contour detection task has been also shown to promote anatomical reorganization of the V1 circuitry, shaping the axonal arbors of neurons representing the trained part of the visual field (Van Kerkoerle et al., 2018).

#### 2.1.2 Changes in higher-order visual areas

An increasing number of studies have begun to question the traditional notion of a vPL process solely relying on plastic rearrangements occurring in early visual areas. Indeed, visual perceptual learning (vPL) appears to involve not only localized changes within specific visual areas but also distributed neural and plastic reorganization that reshapes the broader visual network. Yang and Maunsell (2004) provided evidence for vPL-induced changes in monkey middle-order visual areas. After an orientation discrimination training, V4 neurons with receptive fields overlapping the trained location had stronger responses and narrower orientation tuning curves than neurons with receptive fields in the untrained hemifield (Yang and Maunsell, 2004). Since this pioneering evidence, numerous studies have reported changes in the response properties of neurons in monkey V4 in response to vPL. For example, changes in V4 neurons have been dynamically followed during vPL through chronically implanted electrodes (Sanayei et al., 2018). In response to a fine categorization task, V4 neurons increased their ability to represent small contrast differences. Moreover, learning also altered the relationship between signal and noise correlations facilitating downstream decoding (Sanayei et al., 2018).

To date, changes in neuronal response properties associated with vPL training have been documented in several high-order visual areas including, among others, the middle temporal area (Zohary et al., 1994), the lateral intraparietal area (Gold et al., 2008), the inferior temporal area (Adab et al., 2014), the lateral occipital cortex (Kuai et al., 2013), and the fusiform face area (Bi et al., 2014). Notably, training in discriminating simple grating stimuli can change the orientation preference of neurons located in the inferior temporal area, suggesting a crucial involvement of higher-order visual areas even in simple perceptual tasks (Adab et al., 2014).

The effects of vPL on global cortical organization are evident in task-related shifts of stimulus representation from one visual cortex to another. In humans, repetitive transcranial magnetic stimulation (TMS) interference of the left posterior parietal cortex (PPC) impairs feature-difference and signal-to-noise discrimination tasks before training. On the contrary, TMS impairments of the lateral occipital cortex (LO) activity have modest effects. After vPL practice, however, TMS interference has the opposite effect: TMS of the LO impairs discrimination task, while TMS of the PPC ceases to affect the behavioral outcome (Chang et al., 2014). These findings suggest that practice can shift the task representation from PPC to other neurons located in a different cortical area. Another TMS study has revealed that the cortical locus for processing noisy motion signals is shifted from the middle temporal area to the visual area 3 accessory (V3A) after motion direction discrimination (Chen et al., 2016). Similarly, combined results from functional magnetic resonance imaging (fMRI) and TMS proved that the intraparietal sulcus (IPS) becomes engaged only after training, suggesting that even practicing orientation discrimination can globally alter the representation of stimulus features at different cortical levels (Jia et al., 2021).

#### 2.1.3 Toward a brain-wide model for vPL

According to the visual cortical hierarchy theory, the visual scene is analyzed in a feedforward sequence from simple to complex attributes moving from one cortical stage to the next (Felleman and Van Essen, 1991). This view has an important limitation. The functional properties of visual neurons, indeed, are fundamentally dynamic, and the information computed at one cortical stage is sent back to the previous stages to effectively adapt neuronal response according to the environmental features or stored memories (Gilbert and Li, 2013).

In the contest of perceptual tasks, top-down connections affect neuronal tuning, allowing the system to retain stimulus components that are relevant for the task being performed and simultaneously discard task-irrelevant components (Gilbert and Li, 2013). In agreement to this view, several neurophysiological studies suggest that the frontal eye field, a prefrontal cortical region, serves as a source of top-down signals to the area V4 necessary for attentional selection of a target among distractors (Monosov et al., 2008; Zhou and Desimone, 2011).

An emerging line of research suggests that top-down processing has a crucial role in vPL. Cortical mechanisms underlying vPL are currently supposed to involve more complex changes than the mere plastic remodeling of sensory maps lying within early cortical areas. In support to this view, several studies have shown that generalization of learning may occur when multiple tasks (Zhang et al., 2010; Zhang and Yang, 2014; Szpiro and Carrasco, 2015) or more stimulus categories (Green et al., 2015) are involved, and when exogenous attention is directed toward the trained stimuli (Astle et al., 2014). In addition, other findings have shown that C1 can also be top-town modulated (Kelly et al., 2008; Rauss et al., 2009), and C1 changes have been linked to learning transfer, leading to the hypothesis that these changes could at least partially result from a top-down modulation (Zhang et al., 2015). Until recently, however, C1 changes have been traditionally studied to support the prime role of early visual stages in vPL (Pourtois et al., 2008; Bao et al., 2010).

The importance of top-down influence in vPL is further corroborated by the effects that both attention and internal or external feedback can exert on vPL. Selective attention is typically necessary for proper vPL, as it is required not only to enhance the features of trained stimuli but also to suppress irrelevant ones (Vidnyánszky and Sohn, 2005). For example, internal feedback can improve orientation discriminability, provided that it can repeatedly induce activation patterns in early visual stages that are similar to those activated by real visual stimulation (Shibata et al., 2011). Repeatedly imagining different vPL tasks can improve behavioral performance without actual physical stimulation (Tartaglia et al., 2009, 2012). Also, it has been proven that discrimination performance can change from childhood to adulthood due to changes in the mechanisms of selective attention in vPL learners at different developmental stages (Frank et al., 2021). Regarding external feedbacks, an error signal after each incorrect response can facilitate learning (Shiu and Pashler, 1992; Herzog and Fahle, 1997), whereas random feedback uncorrelated with behavioral responses can impair the learning process (Herzog and Fahle, 1997). The social context can also affect vPL: paired training can indeed result in greater perceptual improvements at a faster learning rate than single training (Zhang et al., 2023).

In a series of elegant papers, the Gilbert’s research group has analyzed the contribution of top-down processing in vPL through a bisection task (Crist et al., 1997, 2001; Li et al., 2004, 2008). Practicing this visual discrimination task leads to behavioral improvements and alters neuronal response properties in monkey V1 (Crist et al., 1997, 2001). Contextual interactions were shown to alter V1 responses even during this simple discrimination bisectional task, which was conventionally supposed to be entirely computed within early visual stages (Ito and Gilbert, 1999; Crist et al., 2001). Remarkably, striking context-related effects were found in monkeys trained to do two different discrimination tasks with the same visual stimulus at the same visual field location; V1 neurons responded differently to the same visual stimulus under diverse experimental conditions (Li et al., 2004). These results led the authors to postulate a critical contribution of top-down processing even for simple vPL phenomena. To specifically dissect the role of top-down integration, in a subsequent study, these authors recorded neuronal response after vPL practice in anesthetized monkeys. Inhalation anesthetics, indeed, appeared to preferentially reduce top-down connections (Lamme et al., 1998; Ku et al., 2011; Jordan et al., 2013; Raz et al., 2014). Previously recorded learning-induced V1 neuronal changes disappeared completely under anesthesia (Li et al., 2008). Similarly, transient chemical inhibition of the monkey middle temporal area impaired perceptual performance on a coarse-depth discrimination task (Chowdhury and DeAngelis, 2008). In humans, a transient interference of the middle temporal area or of the posterior parietal cortex affects the performance in several discrimination tasks (Chang et al., 2014; Chen et al., 2016).

The evidence presented so far suggests that the plastic changes at early visual stages appear insufficient to entirely account for the complexity of vPL. Learning a vPL task may instead rely upon a distributed net of brain-wide plastic rearrangements at different stages of cortical processing rather than entirely depend on changes that tune the activity of V1 neurons to the simple features of trained stimuli (Maniglia and Seitz, 2018). Accordingly, simultaneous recording in the monkey V4 and prefrontal cortex have demonstrated that these two cortical regions show synergic plastic changes over the course of vPL training (Jing et al., 2021). The recruitment of prefrontal neurons was also observed in the mouse brain, where neurons have proven crucial in the completion of a behavioral task (Wang et al., 2023). Within the visual system, instead, simultaneous recordings in monkey V1 and V4 have unraveled interdependent bottom-up and top-down processes that operate synergistically to enhance the internal representation of the practiced stimulus feature, resulting in parallel increments of coded information in both visual areas (Chen et al., 2014).

Thus, several recent studies suggest that perceptual improvements, as well as the associated brain changes, arise from specific interactions between stimulus-driven bottom-up processes and goal-directed top-down influences (Watanabe and Sasaki, 2015; Li, 2016; Maniglia and Seitz, 2018; Astorga et al., 2022). Therefore, it is reasonable to hypothesize that vPL, just like perception *per se*, might be mediated by several processes engaging cortical areas belonging to different hierarchical stages that are specialized for sensory processing, attentional deployment, and decision making (Maniglia and Seitz, 2018). In this scenario, the neuronal mechanisms underlying vPL may be described as a widespread reorganization of the visual system mediated not only by an improved representation of the trained stimulus within early visual areas, but also by changes in top-down integration.

### 2.2 Anatomo-functional interactions between top-down connections and local circuits

Although several emerging studies support the crucial involvement of top-down integration in vPL, the anatomical framework mediating the interaction between feedback projections and local cortical circuits in early visual areas remains elusive.

One possibility is that functional changes associated with vPL are conveyed by top-down projections re-entering posterior visual areas and synapsing on horizontal connections (Gilbert et al., 2001; Gilbert and Li, 2012, 2013). These connections extend from V1 pyramidal neurons, whose axons travel for long distances parallel to the cortical surface, connecting neurons with separated receptive fields but with similar orientation preference (Gilbert and Wiesel, 1979, 1983, 1989; Rockland and Lund, 1982). Their extent and high specificity make horizontal connections ideally suited conveyors of top-down integration. For their anatomical properties, horizontal connections could mediate input selection over the course of vPL. By selecting relevant components of the trained stimulus, horizontal connections could allow neurons located in early stages of visual processing to encode high-order information relevant to the vPL task being performed (Piëch et al., 2013). The selectivity of the horizontal connections is indeed dynamic and can be modulated according to task demands, providing a framework for integrating complex stimulus attributes re-entering posterior visual stages through top-down connections. Sprouting and pruning of horizontal connections have been detected in the trained part of monkey V1 over the course of vPL (Van Kerkoerle et al., 2018). To our knowledge, however, direct evidence of the recruitment of horizontal connections by feedback connections during vPL is still missing.

Top-down integration could also require changes in the inhibitory system (Bastos et al., 2012; Gilbert and Li, 2012; Nurminen et al., 2018; Vangeneugden et al., 2019). Suppression of task-irrelevant features is indeed associated with changes in GABAergic inhibition (Frangou et al., 2019). At the cellular level, inhibitory neurons show experience-dependent remodeling, both in their dendrites (Chen et al., 2011) and in their axon (Marik et al., 2014). Notably, activation of specific interneurons is sufficient to improve perceptual discrimination (Lee et al., 2012) and vPL practice correlates with recalibration of the excitatory/inhibitory balance (Baroncelli et al., 2012).

Although it is known that all the major subtypes of interneurons are targeted by feedback projections entering V1 (Gonchar and Burkhalter, 2003), the contribution of the inhibitory system to vPL remains to be addressed. Feedback projections primarily target parvalbumin-positive (PV+) interneurons, the largest proportion of inhibitory neurons (Tremblay et al., 2016; van Versendaal and Levelt, 2016). Most PV+ neurons have wide dendritic trees and predominantly innervate pyramidal neurons (Somogyi et al., 1983). PV+ interneurons are indeed known for the precise control that they can exert on the spike timing of neighboring excitatory neurons (Pouille and Scanziani, 2001; Wehr and Zador, 2003). PV+ interneurons might therefore be recruited during vPL to regulate the firing of the specific cluster of pyramidal neurons engaged in the perceptual task, improving the evoked response of selected excitatory neurons within V1 electrical noise.

Nonetheless, some results suggest that the synapses between feedback projections and PV+ interneurons are small and located on thin dendrites (Gonchar and Burkhalter, 1999; Yamashita et al., 2003). The excitation provided by feedback projections onto PV + interneurons might therefore be insufficient to precisely drive the activity of primary visual neurons in response to top-down information (Shao and Burkhalter, 1999). Feedback projections innervate also inhibitory interneurons located in V1 superficial layers. A potential role in vPL for the major inhibitory subtype in superficial layers – the vasoactive intestinal peptide-positive (VIP+) interneurons – cannot be excluded. Such an hypothesis could align with the emerging notion that superficial cortical layers are crucially implicated in mediating the integration of top-down interactions (Ibrahim et al., 2021; Schuman et al., 2021). VIP+ interneurons are bipolar cells specialized in inhibiting the activity of other interneuron subtypes (Tremblay et al., 2016; van Versendaal and Levelt, 2016). In this scenario, top-down integration might be mediated by specific rearrangements within the inhibitory system that eventually regulate the activity of selected pyramidal neurons during vPL.

**FIGURE 2:**
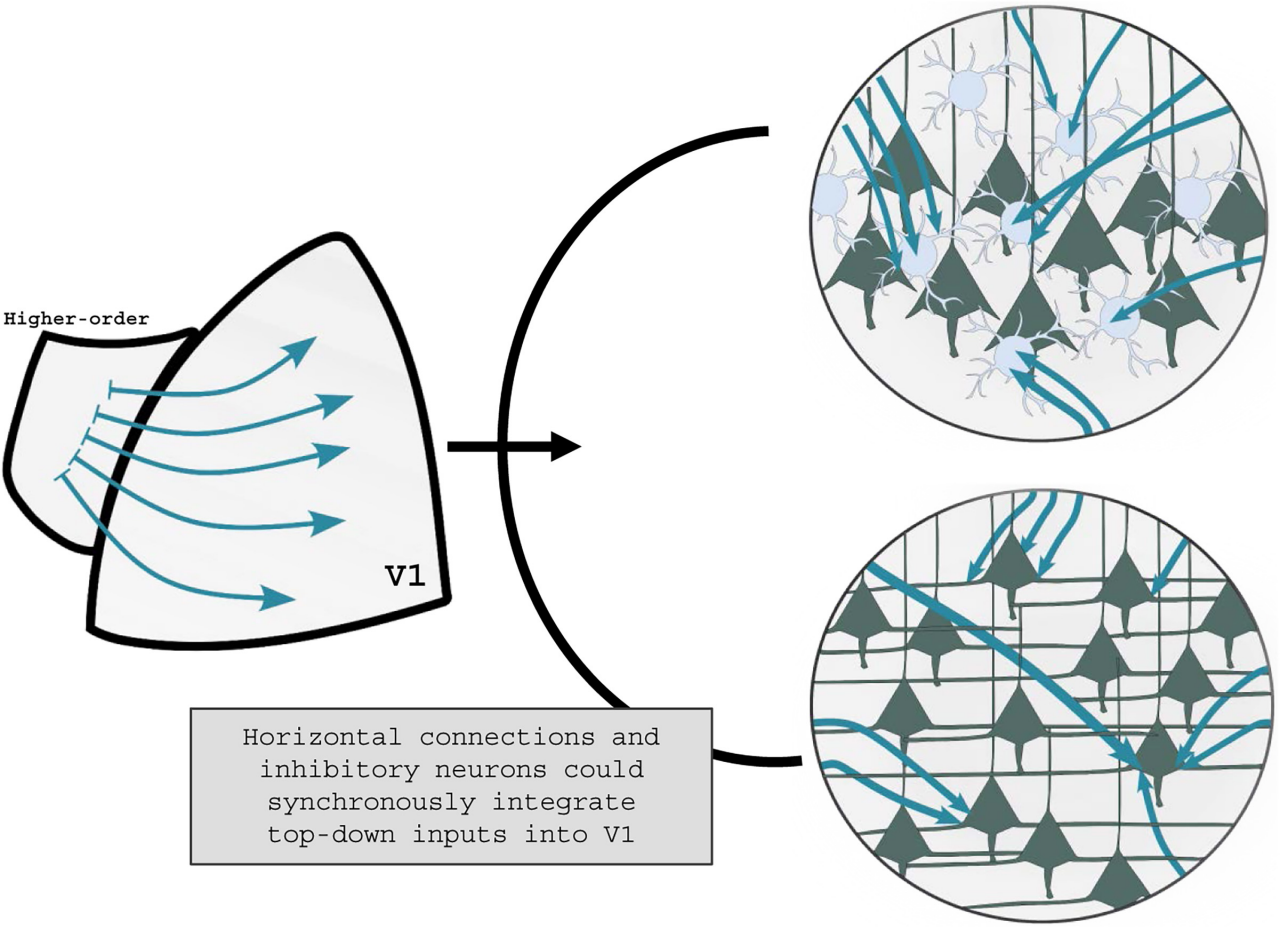
Visual perceptual learning shaping of top-down communication. It is yet unknown how the activation of higher-order visual areas during vPL can tune V1 activity. On one end, top-down projections could affect the V1 inhibitory tone by activating specific subclasses of GABAergic interneurons **(top panel)**. On the other end, V1 horizontal connections could be selectively recruited during vPL, with resultant changes in the V1 excitatory tone **(low panel)**. It is plausible to hypothesize that in response to vPL higher-order visual areas recruit both GABAergic interneurons and horizontal connections, leveraging the excitatory/inhibitory balance into V1 circuitries.

It is worth noticing, however, that top-down integration in vPL could be synchronously mediated by the synergic activity of excitatory and inhibitory drives. These plastic changes are not mutually exclusive processes: excitatory and inhibitory circuits could both affect the signal to noise ratio within V1, ultimately improving perceptual discrimination (Figure 2).

### 2.3 vPL in rodents

Over the last decades, rodents have become invaluable models to study visual processing over the last two decades. Anatomical studies have observed multiple analogies between mouse and primate visual systems (Glickfeld and Olsen, 2017). Behavioral evidence has also shown that mice exhibit a wide range of visually-guided complex behaviors (Prusky et al., 2000; Morcos and Harvey, 2016; Dickson et al., 2017). Moreover, the availability of genetic and molecular tools in rodents enable precise investigation of cellular, circuitry and inter-cortical interactions.

A well-studied form of experience-dependent response enhancement is the stimulus-selective response potentiation (SRP). SRP is the long-lasting response enhancement occurring in the V1 of awake mice repeatedly exposed to visual stimuli (Frenkel et al., 2006). Alike vPL, this phenomenon is selective for trained stimulus features (such as orientation, spatial frequency, and contrast), shows a progressive onset over training sessions, and it can occur in both juvenile and adult mice (Cooke and Bear, 2012; Montgomery et al., 2022). SRP acquisition is tightly linked to the expression of a specific class of ionotropic glutamate receptors (NMDAR): local infusions of NMDAR antagonists or the genetic ablation of NMDAR subunits, indeed, prevent SRP acquisition (Frenkel et al., 2006; Montgomery et al., 2022). Notably, several studies proved that inhibitory neurons are crucially involved in SRP generation (Heynen and Bear, 2001; Kaplan et al., 2016; Montgomery et al., 2022). For example, chemogenetic inactivation of PV+ interneurons can occlude SRP expression. Selective ablation of NMDARs in these interneurons is sufficient to impair SRP (Kaplan et al., 2016). Even though SRP is reminiscent of vPL, it fails to thoroughly model this form of learning as it lacks the incremental component of vPL. SRP, indeed, is dependent on a passive view of visual stimuli.

**FIGURE 3:**
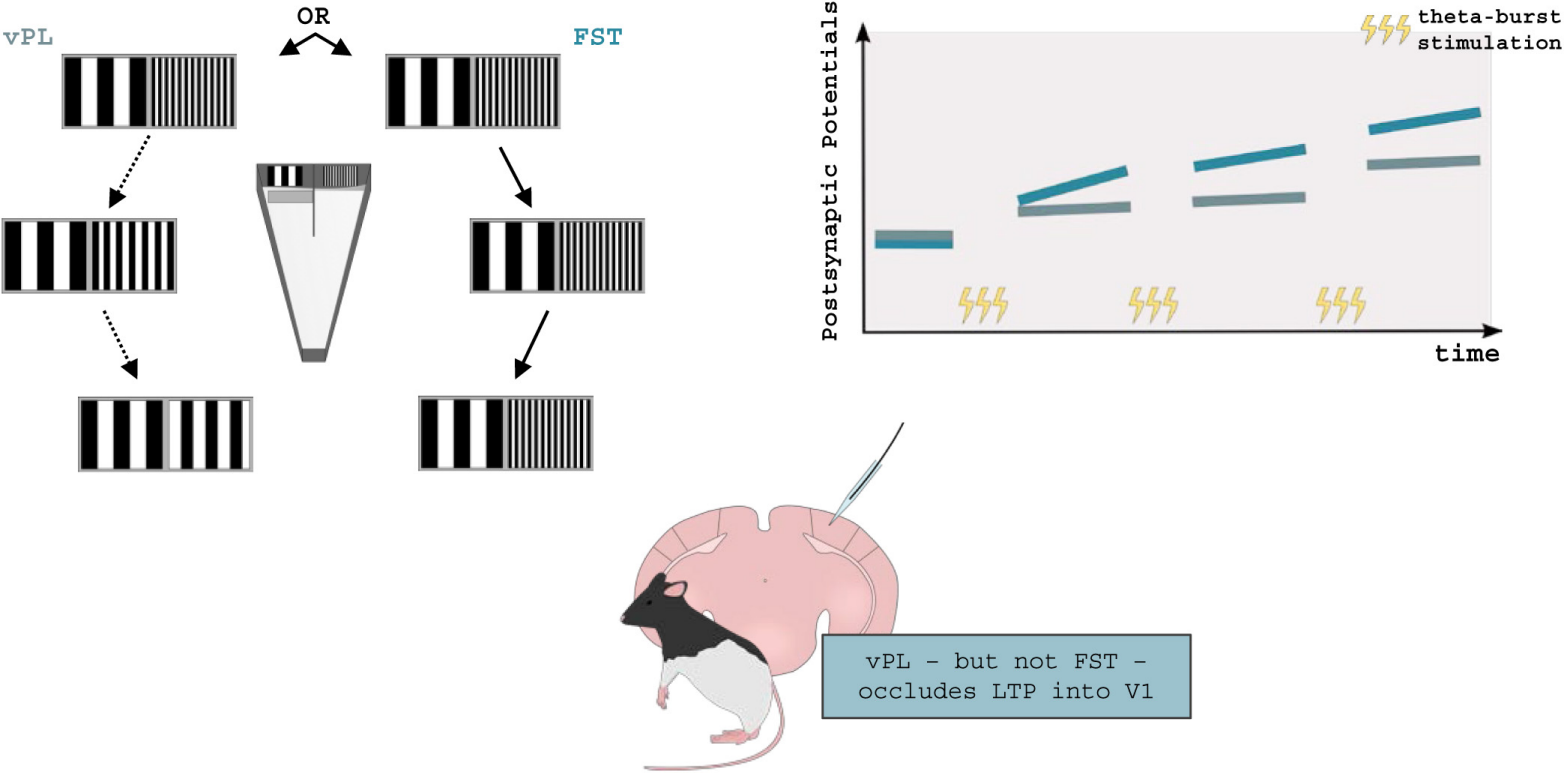
Visual perceptual learning and synaptic plasticity in rodents. vPL can be induced in adult rodents by employing a modified version of the visual water task. Long-Evans rats were required to distinguish between two visual gratings made progressively similar to each other, until a perceptual plateau was reached (vPL rats). In parallel, control rats were only allowed to distinguish between the initial pair of visual gratings First-Step Training (FST) rats. After training, Long-Term Potentiation (LTP) was occluded in vPL but not FST mice suggesting that vPL can increase V1 synaptic efficacy. Adapted from Sale et al. (2011).

As for vPL, few studies have been performed in rodents. A rodent model of vPL was developed by Sale and collaborators (Sale et al., 2011), adapting a discrimination task previously developed by Fiorentini and Berardi (1980). In this task, the animals were asked to discriminate, in an operant two-choice task, two visual gratings with equal contrast but different spatial frequencies. Once the animals learned this simple discrimination, the difficulty of the task was increased by making the two stimuli progressively more similar to each other. Using this task, vPL was shown to result in long-term potentiation (LTP) of V1 connections, a classic cellular mechanism of cortical plasticity. Within one hour from the last vPL trial, LTP recorded *ex vivo* from V1 neurons was occluded in vPL trained rats, both at the level of vertical (layer IV - layer II-III) and horizontal (layer II-III – layer II-III) connections (Figure 3). These results show that plastic changes in synaptic efficacy are induced by vPL in lower-order visual areas of trained animals (Sale et al., 2011). Consistently, it has been proven that vPL can induce structural changes in the dendritic spine density of pyramidal neurons of the rodent V1 (Wang et al., 2016).

#### 2.3.1 The mouse as a prime model to study top-down information in vPL

The mouse brain represents a very promising model to investigate the role of specific intracortical connections involved in vPL, with a focus on top-down projections. In particular, the large availability of advanced techniques makes this animal model extremely suited to probe the role of top-down projections in vPL.

In contrast to the visual system of primates, where V1 is surrounded by a single secondary visual area (V2), the mouse V1 is adjoined by multiple higher-order visual areas (HVAs). In the last 60 years, the number and exact borders of these areas have been redefined and drawn several times, with new evidence emerging from cytoarchitectonic, electrophysiological, or tracer injection experiments (Caviness, 1975; Olavarria and Montero, 1989; Wagor et al., 1980). The current classification of HVAs consists of nine retinotopically organized areas surrounding the mouse V1 (Wang and Burkhalter, 2007). Despite the presence of multiple HVAs, mouse V1 shares its vertical meridian only with one area, the lateromedial cortex (LM), closely resembling the border between V1 and V2 in primates and carnivores. Thus, LM is currently considered as the homologous of the primate V2 (Wang and Burkhalter, 2007).

Recent attempts to understand the functional and structural organization of HVAs support the notion of a mouse visual system clustered in two internally connected subnetworks that are reminiscent of the dorsal and ventral streams seen in primates (Smith et al., 2017; Wang et al., 2012). One subnetwork, constituted by four lateral HVAs, is mainly devoted to process visual stimulus attributes with high spatial frequency (Murakami et al., 2017; Smith et al., 2017), linking the visual system to ventral brain regions implicated in memory and object identification, including temporal association areas and the entorhinal cortex (ventral stream) (Wang et al., 2012). The second subnetwork, constituted by five anteromedial HVAs, is mainly devoted to process visual stimulus attributes with low temporal frequency (Smith et al., 2017), linking the visual system to dorsal brain regions implicated in spatial movement and navigation, including the retrosplenial, anterior cingulate, and second motor cortices (dorsal stream) (Wang et al., 2012). The transition between ventral and dorsal streams is set by the anatomical border dividing LM from the anterolateral cortex (AL) (Wang et al., 2011), which are the first and second HVAs in terms of connections with V1 (Wang et al., 2012).

The current knowledge on the anatomical organization of feedback projections to V1 is incomplete. According to a retrograde tracer study, top-down projections originate from at least 24 brain areas, but the great majority of these fibers re-enter V1 emerging from HVAs and, mostly, from LM (Morimoto et al., 2021). It is currently held that V1 and HVAs form closed-loop circuits that are topographically organized, with primary visual neurons receiving re-entrant projections from the same higher-order neurons to which they send feedforward inputs (Gonchar and Burkhalter, 1999; Johnson and Burkhalter, 1997).

Few studies started to analyze the role of higher-order visual areas in mouse perception. It has been recently shown that neuronal activity in two different HVAs, namely LM and AL, is required for perceiving even simple visual features, as contrast and orientations (Jin and Glickfeld, 2020). The optogenetic suppression of these two HVAs can indeed decrease the sensitivity for both orientation discrimination and contrast detection in mice trained to perform a go/no-go discrimination task, revealing a crucial involvement of higher-order stages in visual perception (Jin and Glickfeld, 2020). Another study has demonstrated that several HVAs are required for the correct execution of a contrast-change discrimination task. Selective inactivation of these visual areas is sufficient to decrease perceptual performance in trained mice. Remarkably, this behavioral impairment can sometimes be greater than the effects induced by direct V1 inhibition (Goldbach et al., 2021). Visual perceptual experience can also promote phase synchrony entrainment between V1 and LM, but not AL, probably to increase inter-areal communication during learning behavior. Optogenetic LM silencing can consistently decrease post-stimulus V1 oscillatory activity and impair visual discrimination (Tang et al., 2023).

**FIGURE 4:**
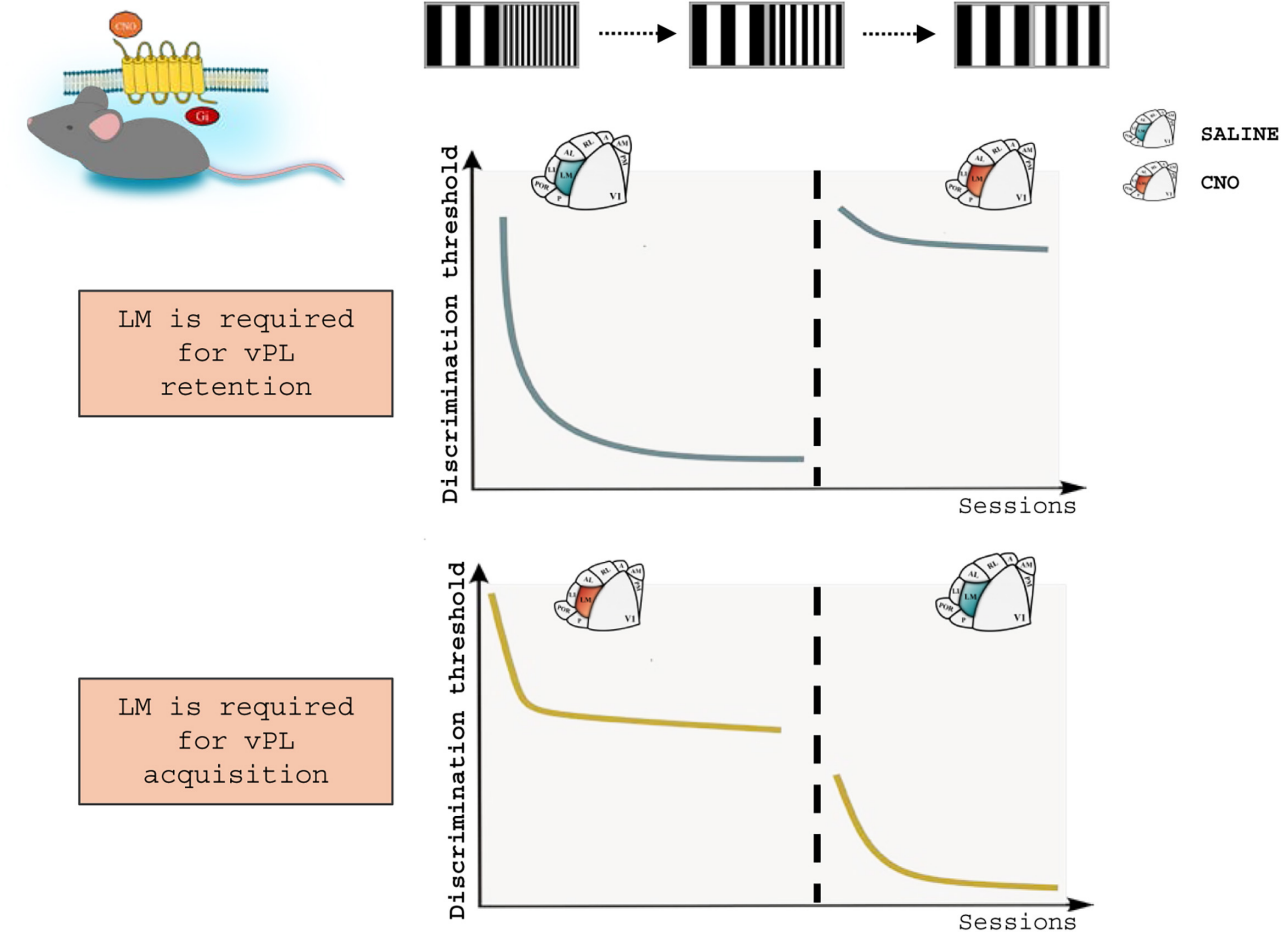
An essential role for top-down integration in vPL. A chemogenetic approach was applied in mice exposed to a vPL task, to analyze the role of LM activity in vPL. **Top:** suppression of LM activity after vPL via the intraperitoneal administration of Clozapine-N-oxide (CNO) leads to the loss of the perceptual improvements acquired during training. **Bottom:** mice administered with CNO during vPL acquisition are unable to complete the task. Noticeably, this impairment can be reversed by restoring LM activity. Top-down activity, then, is involved in both the retention and the acquisition of vPL. Adapted from Consorti et al. (2024).

Higher-order visual areas could also be directly implicated in vPL. The increase in synaptic efficacy recorded in V1 horizontal connections after vPL (Sale et al., 2011), indeed, suggests that higher-order areas could contribute to the acquisition of perceptual improvements as a result of practice. To specifically address this hypothesis, we recently developed a mouse model of vPL (Consorti et al., 2024) analogous to the previously established model for rats (Sale et al., 2011). Among HVAs, we targeted our study on LM, given the homology to primate V2 and its central role in the ventral stream. When LM activity was chronically suppressed via chemogenetic activation before each vPL trial, the animals were completely unable to complete the task, showing a major impairment in vPL acquisition. In a different group of mice, instead, LM activity was suppressed once the perceptual plateau was reached, to probe the role of this HVA in vPL retention. Mice were completely unable to perform the task when LM activity was disconnected from visual circuitry, proving that higher-order neurons are constantly recruited during vPL, even after learning acquisition. Strikingly, we found that the role of LM in vPL is directly mediated by LM to V1 top-down projections. Indeed, similar learning deficits were found when these top-down connections alone were selectively suppressed (Consorti et al., 2024). It appears, then, that vPL strictly depends on the crosstalk between incoming LM top-down projections and V1 neurons (Figure 4).

Higher-order visual areas neurons are thus recruited for the effective completion of vPL. Training relies on the integrated neural activity elaborated in higher-order visual stages, in which information on the context and reward components of the vPL task are likely to be encoded. In parallel, V1 neurons need to retrieve cortical representations of prior information regarding the vPL task, a type of information that can be elaborated at the level of HVA circuitries (Consorti et al., 2024).

## 3 Computational models of hierarchical reweighting

Computational models have played a powerful role in testing the engagement of top-down integration in vPL (Dosher and Lu, 2017). To date, numerous models of vPL have been proposed, including the reverse hierarchy theory (Ahissar and Hochstein, 1993, 2004), the reweighting model of perceptual learning (Dosher and Lu, 1999), the dual plasticity model (Watanabe and Sasaki, 2015), and the dynamic performance-monitoring model (Sotiropoulos et al., 2018). All these computational models are reweighting models (Dosher and Lu, 2017), in which, essentially, learning is accomplished by weight changes in readout connections from lower to higher visual units (Weiss et al., 1993).

A highly influential model in vPL research is the Integrated Reweighting Theory (Dosher et al., 2013; Dosher and Lu, 2017). According to this model, vPL emerges from two independent mechanisms: improved filtering of external noise and reduction of internal noise. The reductions of these two noises are accomplished by the selective reweighting of connections between lower and higher visual areas (Dosher and Lu, 2017). Consistently, top-down connections entering V1 can selectively enhance the contour signals and suppress background elements over the course of contour detection tasks (Piëch et al., 2013; Chen et al., 2014). Training may also refine perceptual representation in higher-order visual stages, which in turn could refine sensory processing in lower visual areas (Kuai et al., 2013).

Another influential model dealing with top-down integration in vPL is the reverse hierarchy theory (Ahissar and Hochstein, 1993, 2004). According to this model, vPL is a top-down guided process. Perceptual improvements stem from a top-down-guided progressive shift in task-relevant information usability from high-order areas to lower-order areas, which have a better signal-to-noise ratio. The learning process is therefore mediated by a cascade of top-to-bottom modifications that enhance task-relevant and prune irrelevant information, leading to an increase in signal-to-noise ratio. The reverse hierarchy theory has received strong support from fMRI studies in human subjects (Furmanski et al., 2004; Sigman et al., 2005; Mukai et al., 2007; Chen et al., 2016). Learning in shape identification, for example, can consistently lead to global activation changes across the entire visual pathway, with earlier stages becoming more active and higher stages becoming less active with training (Sigman et al., 2005).

## 4 Conclusion

We reviewed how perceptual practice rewires brain connectivity and activity, with special emphasis on visual system plasticity. Altogether, the reported findings suggest that PL can promote neural plasticity far beyond the postnatal maturation of brain circuits. Moreover, several studies have indicated that vPL induces plastic remodeling at different stages of visual cortical processing.

The perceptual improvements associated with vPL should then be considered a result of the interaction between early cortical circuits, encoding specific stimulus features, and feedback projections, conveying information on expectations, prior experience and encoded memories, to local cortical circuits. In this context, we have proven that suppressing the activity of the rodent homologous of the primate V2 severely compromises vPL, preventing its acquisition and erasing the learned perceptual skills. Thus, these results indicate that vPL is a multi-regional brain process that engages the projections from higher- to lower-order visual areas.

Despite robust evidence suggesting that vPL rewires top-down networks, a comprehensive understanding of this phenomenon is far from being reached. Further efforts should be made to deepen the current knowledge of how the activity of a higher-order area can locally affect backstream neural processing. A long-standing hypothesis in vPL research suggests that complex percepts are conveyed into V1 through the top-down recruitment of selected horizontal connections. On the other end, novel studies suggest that the prime mediator of higher-to-lower interactions is the inhibitory system. It is reasonable to hypothesize, however, that vPL improvements eventually result from top-down induced changes occurring synergically on both horizontal connections and inhibitory neurons that ultimately tune local networks to task-relevant features, improving the signal-to-noise ratio leveraging V1 excitatory/inhibitory balance.

## Data Availability

The original contributions presented in this study are included in this article/supplementary material, further inquiries can be directed to the corresponding author.
